# Ethiopia’s health sector evolution and WHO’s mandate

**DOI:** 10.2471/BLT.24.292481

**Published:** 2024-11-01

**Authors:** Mekdes D Feyssa

**Affiliations:** a Ministry of Health, Sudan St, 1234, Addis Ababa, Ethiopia.

The World Health Organization (WHO) continues to play a crucial role in shaping global health norms, and its guidance has been instrumental in improving health in Ethiopia over the past two decades. Ethiopia’s health system, with its successes and ongoing struggles, offers a useful lens to examine WHO’s normative leadership and its impact on global health. With emerging threats such as climate change and increasing burden of noncommunicable diseases, reviewing WHO’s role and discussing areas for improvement is timely. 

Guided by WHO’s normative output, in the last two decades Ethiopia has made substantial progress in immunization, maternal and child health, and communicable disease control.^1^ WHO’s recommendations on vaccination strategies and schedules have been central to improving childhood vaccination coverage, introducing life-saving vaccines and achieving milestones such as polio eradication certification in Africa in 2020.^2^^,^^3^ Similarly, Ethiopia’s reduction in maternal mortality, from 871 per 100 000 live births in 2000 to 401 per 100 000 live births in 2017, achieved through improved safe childbirth practices and antenatal care aligned with WHO guidelines, highlights the success of Ethiopia’s Health Extension Programme in extending services to rural areas.^4^^,^^5^ Regarding communicable diseases, WHO’s guidance on treatment and prevention strategies, including antiretroviral therapy for human immunodeficiency virus and malaria control programmes, has considerably contributed to reducing the burden of these diseases.^6^^,^^7^

While WHO’s contributions have been important, the Organization’s normative guidance has had less impact in Ethiopia’s response to certain types of noncommunicable diseases, and health workforce development. Despite WHO’s guidelines on the prevention and management of noncommunicable diseases, the success seen with communicable diseases has not been replicated. *The global action plan for the prevention and control of noncommunicable diseases*, although well intentioned, has proven difficult for Ethiopia to implement due to limited financial and human resources. As a result, hypertension, diabetes and cancer are increasingly prevalent, accounting for almost 40% of deaths in the country.^8^ This disparity underscores a considerable gap between global recommendations and local capacity.

Furthermore, WHO has emphasized the need for a strong health workforce, yet Ethiopia continues to face challenges in this area. Although the government has expanded the health workforce from about 46 000 in 2007 to over 160 000 in 2019, a critical shortage of skilled professionals remains. Implementing WHO’s workforce development recommendations, while valuable, has been hampered by ongoing constraints in human and financial resources, health-care infrastructure and high turnover rates of health workers in rural areas. Ethiopia’s health professional-to-population ratio remains one of the lowest globally, at 1.63 per 1000 people, reflecting the need for more tailored, resource-sensitive approaches to health workforce development.^9^ Ethiopia’s experience underscores the importance of adapting WHO’s guidelines such as *Primary health care – now more than ever* to local contexts, as demonstrated by the success of Ethiopia’s Health Extension Programme in addressing the needs of rural communities.^10^ Additionally, adequate financial and human resources, medical supplies and health-care infrastructure are essential for the effective implementation of WHO’s guidelines. Strengthening local capacity and investing in sustainable health worker training initiatives are instrumental for effective implementation and achieving meaningful impact, making these actions crucial for Ethiopia’s ongoing health progress.

Finally, as the global health landscape evolves, WHO’s normative function must adapt, particularly in the realm of digital health technologies and artificial intelligence. Digital health technologies such as telemedicine and mobile health platforms could offer solutions to persistent challenges such as Ethiopia’s shortage of health workers in rural areas. 

Similarly, artificial intelligence holds promise for enhancing diagnostics, personalizing treatments and improving disease surveillance. However, ethical use and equitable deployment are critical concerns, especially in low-resource settings. WHO’s role in setting global standards for the ethical use of artificial intelligence in health will be important to ensure that these technologies benefit all countries. Ensuring that artificial intelligence-driven solutions are adaptable to local contexts will be essential for their effective implementation in countries like Ethiopia.^11^ WHO’s leadership in integrating these technologies into existing health systems is fundamental to ensure they are accessible, scalable and sustainable.^12^
